# *Aspergillus nidulans* Synthesize Insect Juvenile Hormones upon Expression of a Heterologous Regulatory Protein and in Response to Grazing by *Drosophila melanogaster* Larvae

**DOI:** 10.1371/journal.pone.0073369

**Published:** 2013-08-26

**Authors:** Morten Thrane Nielsen, Marie Louise Klejnstrup, Marko Rohlfs, Diana Chinyere Anyaogu, Jakob Blæsbjerg Nielsen, Charlotte Held Gotfredsen, Mikael Rørdam Andersen, Bjarne Gram Hansen, Uffe Hasbro Mortensen, Thomas Ostenfeld Larsen

**Affiliations:** 1 Department of Systems Biology, Technical University of Denmark, Kgs Lyngby, Denmark; 2 J.F. Blumenbach Institute of Zoology and Anthropology, Georg-August-University Göttingen, Göttingen, Germany; 3 Department of Chemistry, Technical University of Denmark, Kgs Lyngby, Denmark; University of Nebraska, United States of America

## Abstract

Secondary metabolites are known to serve a wide range of specialized functions including communication, developmental control and defense. Genome sequencing of several fungal model species revealed that the majority of predicted secondary metabolite related genes are silent in laboratory strains, indicating that fungal secondary metabolites remain an underexplored resource of bioactive molecules. In this study, we combine heterologous expression of regulatory proteins in *Aspergillus nidulans* with systematic variation of growth conditions and observe induced synthesis of insect juvenile hormone-III and methyl farnesoate. Both compounds are sesquiterpenes belonging to the juvenile hormone class. Juvenile hormones regulate developmental and metabolic processes in insects and crustaceans, but have not previously been reported as fungal metabolites. We found that feeding by *Drosophila melanogaster* larvae induced synthesis of juvenile hormone in *A. nidulans* indicating a possible role of juvenile hormone biosynthesis in affecting fungal-insect antagonisms.

## Introduction

Filamentous fungi are capable of synthesizing a wide range of bioactive molecules important for growth and survival in complex and competitive ecological niches [1]–[3]. A substantial number of these metabolites have been found to have beneficial as well as detrimental impact upon human health. Notable examples of both categories include the pharmaceutically important lovastatin and penicillin [4]; and the mycotoxins fumonisin and aflatoxin that cause health hazards and economical losses when they are present in infected crops [5]. With the release of the full genome sequences of several filamentous fungi it has become apparent that the number of predicted secondary metabolite synthases by far exceeds the number of known metabolites [6], [7]. These observations suggest that specific environmental stimuli are required for induction of the majority of secondary metabolites [8]. Despite attempts to identify or mimic these stimuli in order to unravel the secondary metabolism of the model organism *Aspergillus nidulans*, the product of the majority of predicted synthases are still not known [9], [10]. Genetic approaches have been somewhat successful through manipulation of histone methylation [11] or controlled expression of regulatory proteins [12]. As biosynthetic pathways towards secondary metabolites tend to be clustered in the genome [6], [7] regulatory proteins likely to be involved in secondary metabolism may be identified by genomic co-localization. However, the number of successful applications of this approach is limited, possibly because far from all predicted gene clusters contain regulatory proteins. We decided to investigate whether induction of secondary metabolites could be achieved through heterologous expression of regulatory genes from other filamentous fungi using the expression of *A. niger* proteins in *A. nidulans* as a test case. A selection of putative pathway specific regulators was tested for this purpose by expressing the corresponding genes individually from a defined locus using a constitutive promoter [13]. This genetic approach was combined with a screen of several complex media recently demonstrated to influence *A. nidulans* secondary metabolism [14]. This combinatorial approach resulted in the identification of one regulatory protein that strongly induced metabolites not previously reported from *A. nidulans*. Among the induced metabolites were the sesquiterpene hormones methyl farnesoate and insect juvenile hormone-III. Juvenile hormones are required in exact concentrations for correct development of insects and crustaceans [15]–[17] and therefore hold a strong potential as insecticides [18], [19]. To the best of our knowledge, this is the first observation of a fungus with the capacity of synthesizing juvenile hormones. In this manuscript, the biological function of juvenile hormones in *A. nidulans* was addressed through interaction with the saprophagous insect, *Drosophila melanogaster*. We found that when *A. nidulans* was challenged by grazing insects, synthesis of juvenile hormones was induced suggesting that juvenile hormones are part of the fungal defense against invertebrates.

## Results and Discussion

### Procedure for selection of candidate genes

Selection of candidate regulatory proteins associated with secondary metabolism was based on genomic co-localization of gene clusters. We utilized a collection of previously published microarray experiments from *A. niger* grown under diverse conditions [20]–[22] to identify regulatory genes associated with predicted secondary metabolite gene clusters using a recently described co-expression based algorithm [23]. Seven candidate genes associated with predicted gene clusters containing either polyketide synthases or non-ribosomal peptide synthases, were identified (Table 1). All seven putative transcription factors belong to the binuclear zinc finger class of proteins, a class often associated with secondary metabolism in fungi [24]. BLAST analysis [25] using the predicted protein sequences against the annotated *A. nidulans* genome (Aspergillus Comparative Database, BROAD Institute) revealed that only one candidate (fge1_pg_C_4000037) had a potential ortholog (ANID_06396, 62% amino acid identity, Table 1). Genes encoding all seven putative regulators were expressed individually in *A. nidulans* under control of the strong constitutive *PgpdA*-promoter from the defined locus *IS1* [13].

**Table 1:** Candidate genes from *A. niger*.

Strain #	Broad annotation	Transcript ID	Candiate *A. nidulans* homologues	Identity percentage
**NID357**	fge1_pg_C_4000037	38716	ANID_06396, ANID_03269	62%, 27%
**NID358**	e_gw1_4.316	178503	ANID_07346	26%
**NID360**	e_gw1_11.945	188323	ANID_08894	25%
**NID366**	gw1_10.247	123782	None	–
**NID367**	fge1_pg_C_19000192	45823	ANID_11683, ANID_07921	43%, 22%
**NID476**	e_gw1_8.296	184613	ANID_04485	30%
**NID477**	est_fge1_pg_C_150220	54836	None	–

Candidate genes were selected based on co-localization with predicted gene clusters in *A. niger* containing either a polyketide synthase, a non-ribosomal peptide synthase or both. Transcript ID =  Annotion from the DOE Joint Genome Institute (genome.jgi-psf.org), candidate *A. nidulans* homologues  =  Highest scoring potential homologs in *A. nidulans*, Identity percentage  =  amino acid identity percentage.

### Chemical analysis of mutant strains identifies juvenile hormones as metabolites of *A. nidulans*

**Figure 1:**
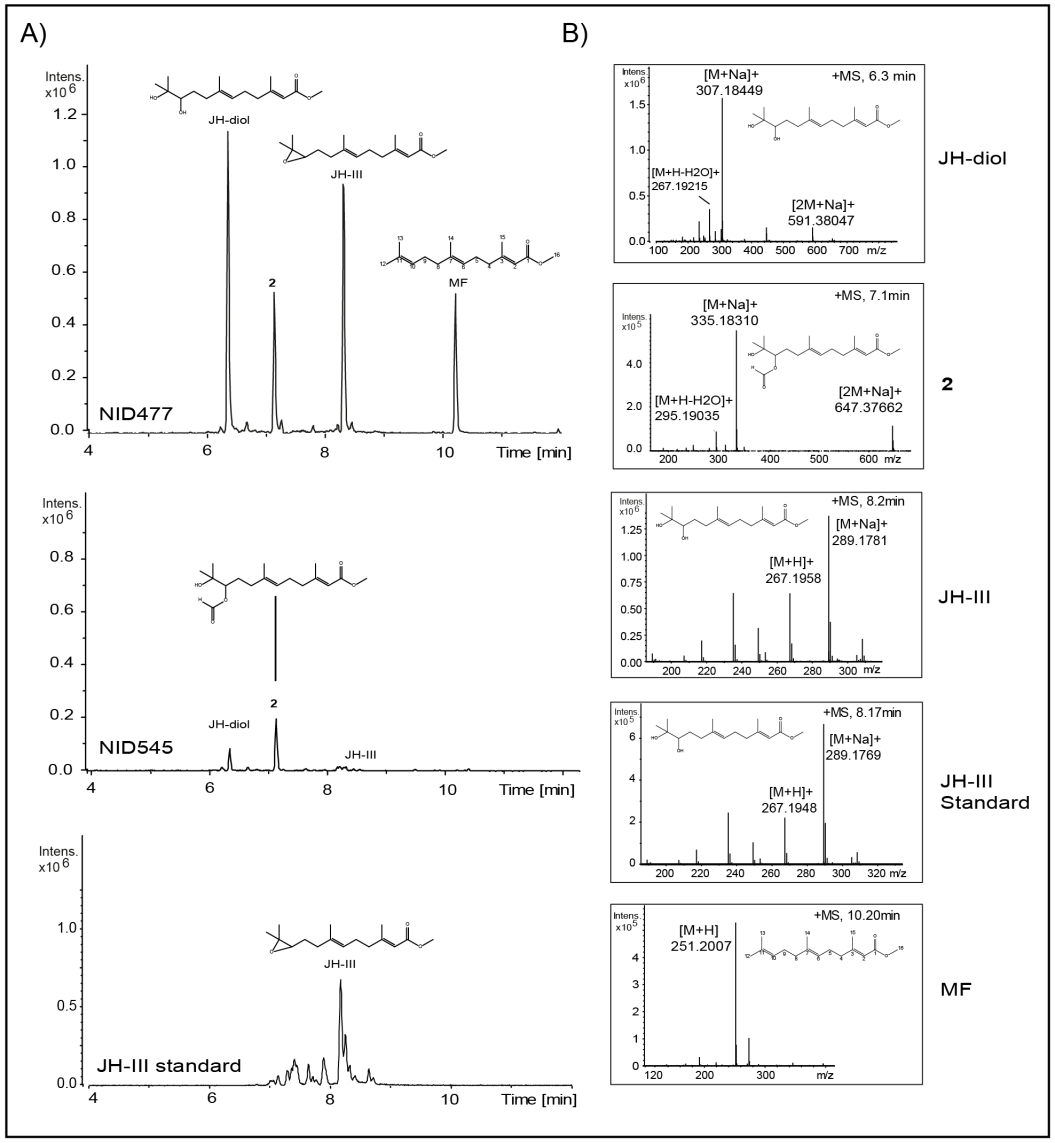
Induction of metabolites by SmrA. A) UHPLC-QTOFMS extracted ion chromatogram of m/z 251 (MF, [M+H]^+^), 289 (JH-III, [M+Na]^+^), 307 (JH-diol, [M+H]^+^) and 335 (X2, [M+H]^+^) recorded in positive mode of extracts from the strain constitutively expressing *smrA* (top) and reference (middle) grown under high salt conditions. Chromatograms are normalized by intensity. Chemical structures of JH-diol, compound **2**, JH-III and MF are embedded above the corresponding signal peaks. Bottom panel depicts extracted ion chromatogram of m/z 289 (JH-III, [M+Na]^+^) of an authentic JH-III standard (65% pure) purchased from Sigma Aldrich. Note that the standard contains several impurities. Panel B): Corresponding mass spectra of JH-diol, compound **2**, JH-III and MF in the mutant strain constitutively expressing *smrA* as well as the authentic JH-III standard. Chemical structure of the corresponding molecule is embedded in each panel.

**Figure 2:**
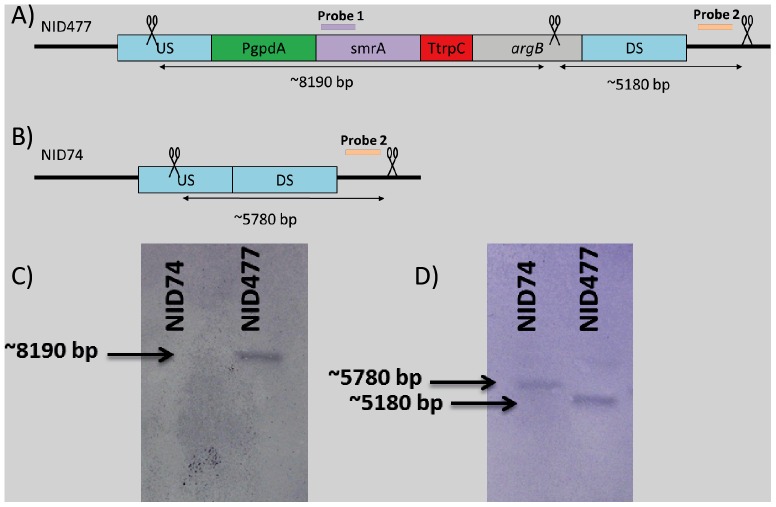
Confirmation of correct integration of smrA in IS1. A) and B): Schematic overview of the HindIII cut sites (indicated with scissors) and the size of the resulting fragments. Purple and orange bars indicate hybridization site for *smrA* probe and locus probe respectively. C): Illustration showing placement of the bands relative to each other. D): Southern blot of NID74 and NID477 digested with HindIII and hybridized with *smrA* probe. E): Southern blot of NID74 and NID477 digested with HindIII and hybridized with locus probe. The illustration is not drawn to scale.

**Figure 3:**
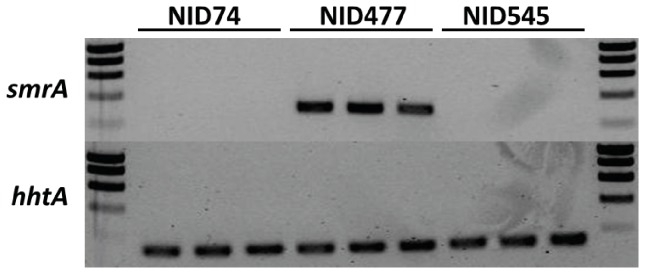
Expression of smrA in NID477 and two control strains. Measuring the expression of *smrA* and the control *hhtA* after cultivating the strains on both MM and CYAs gave the same result. No expression of *smrA* could be detected in the controls (top panel), but only in the NID477 strain.

The resulting mutant strains were grown on minimal glucose media as well as four complex media representing diverse physiological conditions. Metabolite profiles of mycelia extracts were analyzed with liquid chromatography-high resolution mass spectroscopy (LC-HRMS) as well as ultra-high pressure liquid chromatography diode array detection (UHPLC-DAD) and compared to a reference strain that constitutively transcribes the *E. coli* β-galactosidase-gene (*lacZ*) from *IS1* (NID545). Of all combinations of candidate genes and growth conditions, only *est_fge1_pg_C_150220* (annotation from Aspergillus Comparative Database, BROAD institute of Harvard and MIT) propagated under high salt conditions had an immediately appreciable impact on secondary metabolism resulting in increased accumulation of several metabolites not previously reported to be produced by *A. nidulans* (Figure 1A). Hence, we renamed *est_fge1_pg_C_150220* Secondary Metabolism associated Regulatory protein A (*smrA*). The strain that constitutively transcribe *smrA* was denoted NID477, see Table 2. Correct integration of *smrA* into *IS1*, as well as presence of *smrA* mRNA, was confirmed by Southern blot (Figure 2) and quantitative RT-PCR (Figure 3), respectively. Two induced metabolites displaying very similar UV-spectra were isolated from extracts of NID477 and identified by NMR analysis as the sesquiterpenes: methyl (2E,6E)-10,11-dihydroxy-3,7,11-trimethyl-2,6-dodecadienoate (**1**) (JH-diol) [26] and its formylated analogue (**2**). The formylation, however, was subsequently demonstrated to occur during the extraction procedure and **2** is therefore an artificial derivative of **1**. The sesquiterpene **1** (JH-diol) represents the hydrated form of insect juvenile hormone-III (JH-III). This observation prompted us to search for JH-III and related metabolites using targeted LC-HRMS analysis. Indeed, metabolites with accurate masses corresponding to JH-III and the related crustacean hormone methyl farnesoate (MF) [15] were strongly induced in NID477 compared to the reference, NID545 (Figure 1A). Final identification of these metabolites as JH-III and MF was established by comparison of retention time and mass spectra with an authentic standard (Figure 1B), or with a reference spectra (Xcalibur software package, Thermo Scientific), respectively. The discovery of JH-III and MF as metabolites of *A. nidulans* represents to our knowledge the first report of production of invertebrate juvenile hormones in fungi.

**Table 2:** Name and description of fungal strains used in this work.

Strain #	Genotype	Description	Reference
**NID74**	*argB*Δ, *pyrG89, veA1, nkuA*Δ	Parental strain with permanent deletion of *nkuA and argB* to facilitate gene targeting	This study
**NID545**	*argB*Δ, *pyrG89, veA1, nkuA*Δ *IS1::PgpdA-lacZ::argB*	Reference strain with *E.coli lacZ* integrated in *IS1*.	This study
**NID357**	*argB*Δ, *pyrG89, veA1, nkuA*Δ, *IS1:PgpdA:fge1_pg_C_4000037::argB*	Constitutive expression of putative binuclear zinc finger transcrption factor fge1_pg_C_4000037 integrated in *IS1*	This study
**NID358**	*argB*Δ, *pyrG89, veA1, nkuA*Δ, *IS1:PgpdA:e_gw1_4.316::argB*	Constitutive expression of putative binuclear zinc finger transcrption factor e_gw1_4.316 integrated in *IS1*	This study
**NID360**	*argB*Δ, *pyrG89, veA1, nkuA*Δ, *IS1:PgpdA::e_gw1_11.945::argB*	Constitutive expression of putative binuclear zinc finger transcrption factor e_gw1_11.945 integrated in *IS1*	This study
**NID366**	*argB*Δ, *pyrG89, veA1, nkuA*Δ, *IS1:PgpdA:gw1_10.247::argB*	Constitutive expression of putative binuclear zinc finger transcrption factor gw1_10.247 integrated in *IS1*	This study
**NID367**	*argB*Δ, *pyrG89, veA1, nkuA*Δ, *IS1:PgpdA:fge1_pg_C_19000192::argB*	Constitutive expression of putative binuclear zinc finger transcrption factor fge1_pg_C_19000192 integrated in *IS1*	This study
**NID476**	*argB*Δ, *pyrG89, veA1, nkuA*Δ, *IS1:PgpdA::e_gw1_8.296::argB*	Constitutive expression of putative binuclear zinc finger transcrption factor e_gw1_8.296 integrated in *IS1*	This study
**NID477**	*argB*Δ, *pyrG89, veA1, nkuA*Δ, *IS1:PgpdA::smrA::argB*	Constitutive expression of s*mrA* (*est_fge1_pg_C_150220*) integrated in *IS1*	This study

### Biosynthesis of JH-III, JH-diol and MF in *A. nidulans*

Biosynthesis of juvenile hormones is well characterized in insects [27]. Since further elucidation of the potential role of juvenile hormones in fungal-insect antagonisms would benefit substantially from the generation of null mutants, we attempted several homology based strategies for identification of the biosynthetic pathway for juvenile hormones in *A. nidulans*. Initially, BLAST analysis was performed using previously characterized insect enzymes as input, however, no obvious candidates were identified (data not shown). We speculate that the long evolutionary distance between insects and *A. nidulans* may have obscured a common origin, but it cannot be excluded that an alternative biosynthetic mechanism has evolved in fungi. We tested whether the mixed PKS/NRPS gene cluster in which *smrA* is located (*A. niger* transcript ID: 192362, 128601, 191998, 44877, 44878, 44880 and 54837) is conserved in *A. nidulans* and could provide an alternative biosynthetic route, however, the cluster is not present in *A. nidulans* as evidenced by BLAST analysis of individual genes (data not shown). Moreover, SmrA does not have any homologs in *A. nidulans* (Table 1). Thus homology based methods seems to be challenging. We expect that microarray based analysis of the growth condition dependent synthesis of juvenile hormones in NID477 may serve as a more fruitful route for identification of the juvenile hormone synthesis pathway in *A. nidulans*.

### Biological function of Juvenile hormones in *A. nidulans*

Fungal secondary metabolites are known to play an important role in fungal-insect interactions [2], [3]. Moreover, the role of juvenile hormones in regulating processes of insect metamorphosis, reproduction and metabolism are well described [16], [17]. We therefore hypothesized that the biological function of juvenile hormones in *A. nidulans* is related to interaction with insects. It is known, that timing and dosage of insect exposure to juvenile hormones is crucial for correct development, with fatal consequences of both under- and overexposure [16]. Consequently, synthesis of JH and MF could be employed as a defense mechanism in *A. nidulans* and such a strategy has been demonstrated for the plant *Cyprus iria* [28]. We pursued two experimental lines of evidence in order to test our hypothesis; 1) analysis of the spatial distribution of JH and MF and 2) conducted confrontation experiments between *A. nidulans* and larvae of the saprophagous insect *Drosophila melanogaster*.

### Distribution of JH-III, JH-diol and MF

**Figure 4:**
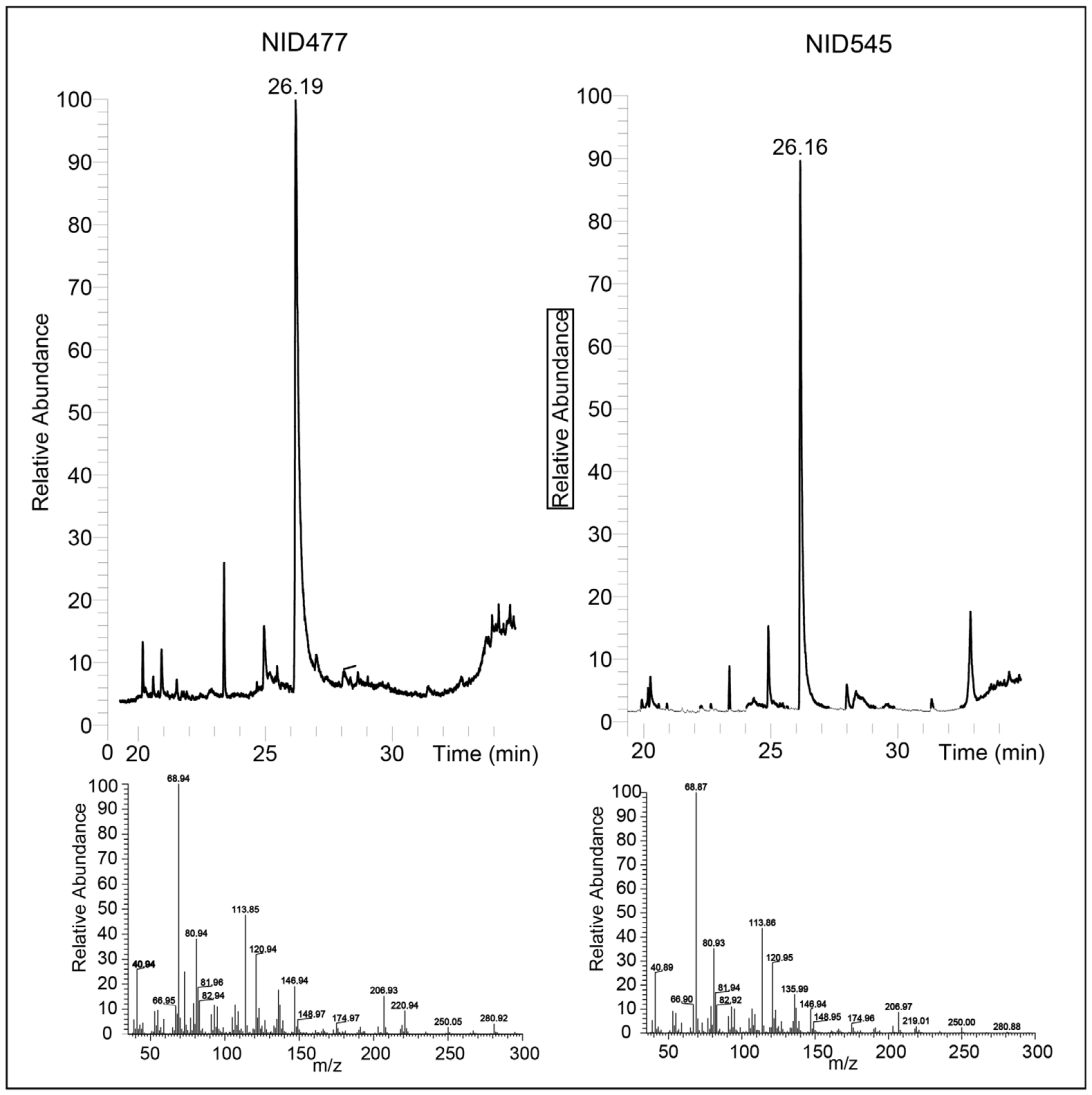
Excretion of MF by *A. nidulans*. Top panel: Total MS chromatogram of the collected volatiles from the *smrA* expressing strain and the reference. Bottom panel: Mass spectrum of the compound eluting at 26.19 minutes. The compound was identified as MF by comparison to the metabolite library of the Xcalibur software package (Thermo Scientific).

The metabolite composition of growth media extracts and collected volatiles of NID477 and the reference, NID545, grown under juvenile hormone stimulating conditions was analyzed by LC-HRMS and gas chromatography mass spectroscopy (GC-MS), respectively. None of the three terpenes were detectable as extracellular metabolites in the growth media. JH-III and JH-diol were also undetectable among the volatiles whereas MF constituted a major metabolite in the volatile fraction of both strains (Figure 4). Taken together with the presence of JH-III and JH-diol in mycelia extracts (see above), we conclude that JH-III and JH-diol are maintained intracellularly in the mycelium. Therefore, insects will ingest juvenile hormones upon foraging on *A. nidulans* which may disturb the careful balance of juvenile hormone dosage.

### *D. melanogaster* larvae induce JH-III synthesis upon grazing

**Figure 5:**
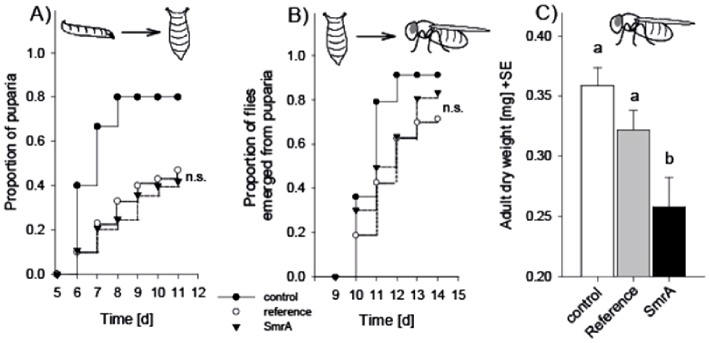
Influence on D. melanogaster larva-to-adult development. Panel A): Proportion of *D. melanogaster* larvae that reached the pupal stage as a function of fungal treatment (mold-free control, NID545 or NID477) and time. Panel B): Proportion of flies that emerged from puparia as a function of fungal treatment and time. Panel C): Dry weight of emerged flies as a function of fungal treatment. Different letters indicate statistically significant differences between treatment following a one-way Analysis of Variance (F_2,38_ = 6.652, p = 0.003) and Holm-Sidak pair-wise comparison. n.s. not significant.

**Figure 6:**
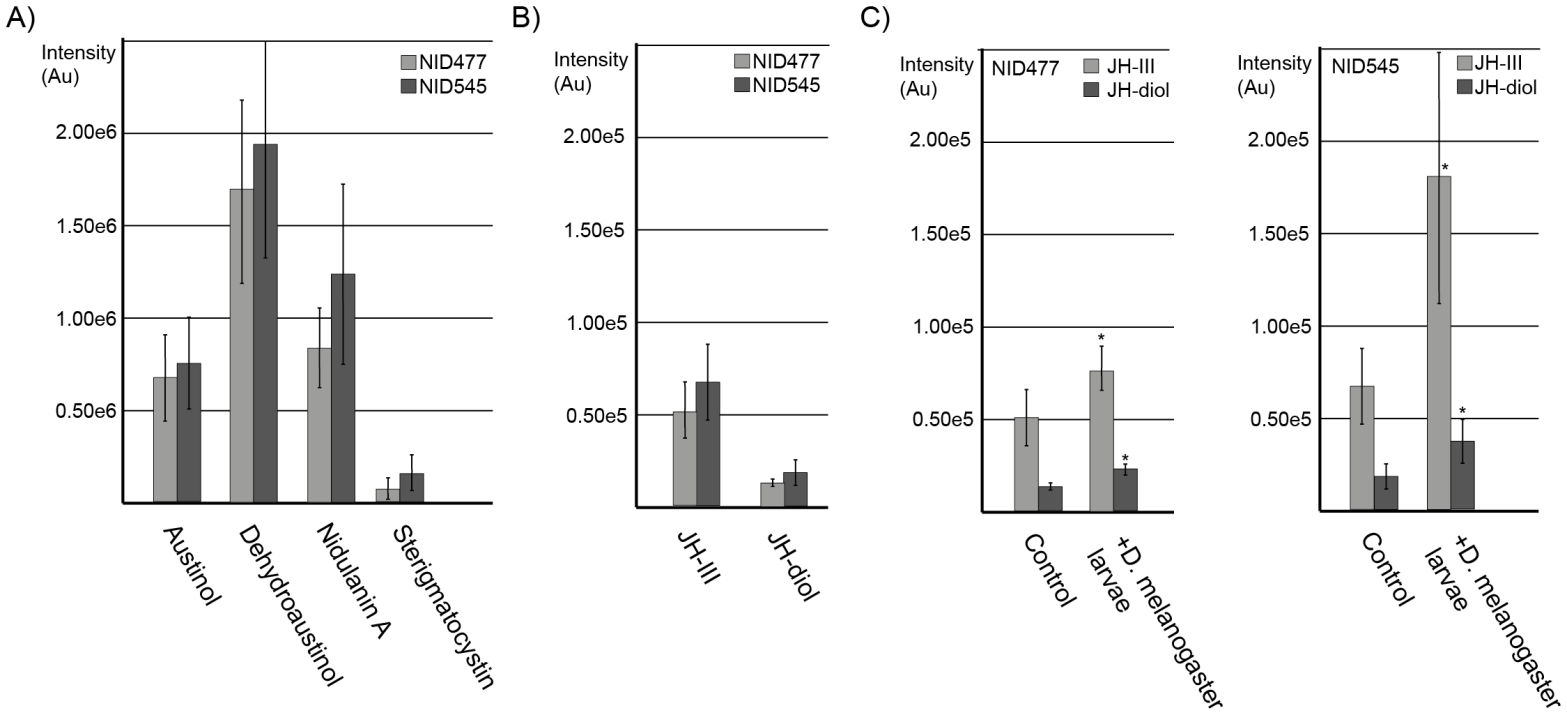
Insect grazing induced alterations of secondary metabolites in *A. nidulans*. Quantification of secondary metabolites: JH-III, JH-diol, austinol, dehydroaustinol, nidulanin A and sterigmatocystin from LC-HRMS analysis. For each metabolite, columns display the average and error bars the standard deviation. Statistical analysis was performed with pair-wise comparisons using the student's t-test. Panel A) comparison of austinol, dehydroaustinol, nidulanin A and sterigmatocystin levels in NID477 and NID545. Panel B) comparison of JH-III and JH-diol levels in NID545 and NID477. Panel C) *D. melanogaster* feeding significantly increases accumulation of JH-III (p-values; 0,0288 and 0,00723) and JH- diol (p-values; 0,0006 and 0,02415) in NID477 and NID545, respectively.

*D. melanogaster* was chosen for the confrontation experiments since the versatile role of juvenile hormones in *D. melanogaster* development is well documented [29] and since patterns of interaction between *A. nidulans* and *D. melanogaster* larvae have been described previously [30]. The confrontation experiments were initially performed under the conditions where SmrA stimulated JH-III and MF synthesis. However, the high salt content in the media (5% NaCl) caused severe larval mortality even in mock free controls (data not shown). We therefore decided to perform the experiments under less stressful conditions (standard *Drosophila* medium, [30]). In this experiment, the fitness of grazing *D. melanogaster* larvae was not significantly different between NID545 and NID477 on two of three parameters evaluated (Figure 5). However, flies emerging from the NID477 treatment displayed a significant decreased dry weight, indicating a negative impact of NID477 on *D. melanogaster* fitness compared to NID545. We therefore performed a metabolite analysis of fungal extracts produced from the two strains in the presence or absence of larvae in order to correlate the observed effect with differences in the metabolite profile. When NID545 and NID477 were grown on standard *Drosophila* medium most of the detectable secondary metabolites (austinol, dehydroaustinol, nidulanin A and sterigmatocystin) did not differ significantly between the two strains (Figure 6A). Importantly, JH-III and the JH-diol were detected in both strains, but was not significantly induced in NID477 on this medium (Figure 6B). These observations are in agreement with the results obtained in the initial screening of the NID477 strain, which revealed that the induction of secondary metabolites was highly condition dependent. Interestingly, comparison of the metabolite profiles obtained from NID477 and NID545 strains grown in the presence or in the absence of grazing *D. melanogaster* larvae demonstrated that the presence of the insects significantly increased the level of both JH-III and JH- diol irrespective of the strain background (p-values JH-III; 0,0288 and 0,00723 and JH-diol; 0,0006 and 0,02415 for NID477 and NID545, respectively, Figure 6C). Curiously, JH-III accumulated to higher levels in NID545 than in NID477 (p-value <0,025). Perhaps, this reflects that when the natural induction of JH-III takes place, the contribution from the presence of the heterologous transcription factor SmrA is detrimental to JH-III biosynthesis. A simple model could be that the natural *A. nidulans* transcription factor and SmrA bind in a competitive manner to the promoters of the genes involved in JH-III biosynthesis and that activation is less efficient when SmrA is present. We consider it likely that constitutive expression of the SmrA transcription factor has numerous other effects on *A. nidulans* that is not reflected in our metabolite analysis, and that collectively these effects cause the observed decrease in *D. melanogaster* fitness. However, the induction of juvenile hormones upon insect feeding, taken together with the well-established involvement of juvenile hormones in insect development and physiology, strongly suggest that JH-III do impact the relation between insects and *A. nidulans*.

### Perspectives

The findings of this manuscript indicate that juvenile hormones represent previously overlooked compounds in chemical interactions between *A. nidulans* and insects. In addition, the ability of *A. nidulans* to synthesize juvenile hormones provides the potential for a bio-based source for juvenile hormone production in cell factories. Juvenile hormones are considered to be among the most potent and promising insecticides due to their high specificity and efficiency [18], [19]. Moreover, as *A. nidulans* releases the juvenile hormone MF to the environment, downstream purification of MF would be simple, as MF could be collected from the volatiles as described previously for other sesquiterpenes [31]. Finally, the findings in this manuscript underline how manipulation of regulatory proteins, systematic variation of physical parameters as well as insect-fungus confrontation systems may be valuable tools for modifying fungal secondary metabolite profiles. The latter approach has the advantage of providing clues to biological function of metabolites. A similar approach simulating bacterial-fungal interactions has previously been successful in identifying novel metabolites in *A. nidulans* [32] indicating that this more biological approach may constitute a promising route for future studies.

## Materials and Methods

### Strains and media

*Escherichia coli* strain DH5α was used to propagate all plasmids. All *A. niger* genes were amplified from strain ATCC1015. The *A. nidulans* strain NID74 (*argBΔ, veA1, pyrG89, nkuAΔ*) was used as background strain for all transformations as it allows gene targeting with the *argB* marker due to a complete deletion of the *A. nidulans argB*-open reading frame. NID74 was generated from NID1 (*argB2, veA1, pyrG89, nkuAΔ*) using the fusion PCR technique essentially as described previously [33]. NID545 (*argB*Δ, *pyrG89, veA1, nkuA*Δ, *IS1::PgpdA-lacZ-TtrpC::argB*) was used as reference strain for metabolite analysis. Genotypes of all strains are summarized in Table 2. All *A. nidulans* strains were propagated on solid glucose minimal medium (MM) prepared as described by Cove [34], but with 1% glucose, 10 mM NaNO_3_ and 2% agar. MM was supplemented with 10 mM uridine (Uri), 10 mM uracil (Ura), where required. Complex media used for chemical analysis were prepared as described by Frisvad and Samson [35] and supplemented with 10 mM uridine and 10 mM uracil.

### PCR, USER cloning and *A. nidulans* strain construction

USER cloning compatible PCR products were amplified with 30 PCR cycles in 50 µl reaction mixtures using proof-reading PfuX7 polymerase [36]. USER vectors were denoted according to the nomenclature introduced by Hansen et al [13]. Putative *A. niger* genes were amplified from *A. niger* genomic DNA, USER cloned into pU1111-*IS1*, and transformed into *A. nidulans* as described previously [13]. In order to generate the NID545 reference strain, the *E. coli lacZ* gene was cloned into a pU1014-*IS1* vector generating pU1011-*IS1*:*lacZ* which was transformed to a pU1110-*IS1-lacZ* vector by insertion of *A. nidulans gpdA* promoter in the AsiSI/Nb.BtsI cassette. All expression plasmids were verified by sequencing. Gene targeting events were verified in all *A. nidulans* transformants by analytical PCR as described previously [13]. Table 3 summarizes the PCR primers used in this study. In addition, NID477 was confirmed by Southern blotting as described in [37]. For each Southern blot 2 µg genomic DNA was digested with HindIII. Two probes for detecting insertion of the *smrA* gene into *IS1* were generated by PCR. Specifically, primers JBN X66 and JBN X67 were used to generate Probe 1, a 896 bp fragment of *smrA* using genomic DNA from *A. niger* as template, and primers JBN X64 and JBN X65 were used to generate Probe 2, a 948 bp fragment at the *IS1* locus using genomic DNA from *A. nidulans* as template, see Figure 2. The probes were labeled with Biotin-11-dUTP using the Biotin DecaLabelTM DNA Labeling kit (Fermentas). Detection was performed with the Biotin Chromogenic detection kit (Thermo scientific).

**Table 3:** PCR primers used in this study.

Primer pair (fw/rv)	Forward primer	Reverse Primer	Description
JBN 2QQ/3QQ	GCCAAGTGGTGGAATGCG	gatccccgggaattgccatgCAACACTATCGCATACTCTCC	Amplifies 2 kb upstream region from a*rgB* (*ANID_04409*) for fusion PCR
JBN 4QQ/5QQ	aattccagctgaccaccatgCCGATCACGTAAAAGCCTGTTAG	CAGGGCGTGGGAGATAGC	Amplifies 2 kb downstream region from *argB* (*ANID_04409*) for fusion PCR
JBN 5A/2K	catggcaattcccggggatcTGGATAACCGTATTACCGCC	GGAAGAGAGGTTCACACC	Amplifies 5′ *A. fumigatus pyrG* sequence including 300 bp direct repeat and native promoter for fusion PCR
JBN 4Q/2B	TGATACAGGTCTCGGTCCC	catggtggtcagctggaattTGCCAAGCTTAACGCGTACC	Amplifies 3′ *A. fumigatus pyrG* sequence including 300 bp direct repeat and native terminator for fusion PCR
JBN 2QQ/2K	GCCAAGTGGTGGAATGCG	GGAAGAGAGGTTCACACC	Amplifies a*rgB* (*ANID_04409*)::*A. fumigatus pyrG* upstream gene targeting fragment
JBN 4Q/5QQ	TGATACAGGTCTCGGTCCC	CAGGGCGTGGGAGATAGC	Amplifies a*rgB* (*ANID_04409*)::*A. fumigatus pyrG* downstream gene targeting fragment
Motni 165/185	cgtgcgauGCAGTGAGAGCGATCGCAGACACTGCATGACCATGATTACGGATTC	cacgcgauTTATTTTTGACACCAGACCA	Amplifies the *E. coli lacZ* ORF for cloning into an AsiSI/Nb.BsmI casette. Forward primer introduces an upstream AsiSI/Nb.BtsI USER cloning casette
Motni 355/354	agagcgauTAAGCTCCCTAATTGGCCC	tctgcgauGCGGTAGTGATGTCTGCTCA	Amplifies 0,5 kb of the *gpdA* promoter for cloning into an AsiSI/Nb.BtsI
287/288	agagcgauATGTTCGTCGCTCCGACGCTT	tctgcgauTCAGAATAAATTGCTTGGAAC	Amplifies the putative ORF of *A. niger* gene *fge1_pg_C_4000037*
289/290	agagcgauATGCGCCATCGACTCACAAA	tctgcgauTTAATAGAGAGCCCATCGC	Amplifies the putative ORF of *A. niger* gene *e_gw1_4.316*
297/298	agagcgauATGAGATCCTCCCAGTCCAA	tctgcgauTCAGCATCCATGCACATGAG	Amplifies the putative ORF of A. niger gene *e_gw1_8.296*
299/300	agagcgauATGAGTCCCGTGTCTGGCCA	tctgcgauTTACATATTCCATGCAAACGA	Amplifies the putative ORF of *A. niger* gene *e_gw1_11.945*
303/304	agagcgauATGGTCTACTGCGGTCGCC	tctgcgauCTATCCACTTAATGGTGGAC	Amplifies the putative ORF of *A. niger* gene *est_fge1_pg_C_150220*
305/306	agagcgauATGAACAGTGAACGAAAGCT	tctgcgauCTATGGATTGGCCATAACCT	Amplifies the putative ORF of *A. niger* gene *gw1_10.247*
307/308	agagcgauATGGATTTGAAACAGAAAGTG	tctgcgauCTATCCTTCGAGAACCTCTT	Amplifies the putative ORF of *A. niger* gene *fge1_pg_C_19000192*
BGHA163/502	GGTCTACTCCCCTGCGTCTA	AGGGAGGCCTTGTCGGTCTT	Check primers for integration in *IS1*. Amplifies the junction between *A. nidulans* chromosome 1 and the *gpdA* promoter
BGHA98/162	GGTTTCGTTGTCAATAAGGGAA	GTTCAGGGTGACGGTGAGAG	Check primers for integration in *IS1*. Amplifies the junction between *A. nidulans* chromosome 1 and the *argB* marker gene
JBN X64/X65	GAACGACGGAACTGTGCTC	CTGCACAATAAGCCACGC	Detection of *smrA* by Southern blot
JBN X66/X67	ATGGTCTACTGCGGTCGC	CGAGACCGATGACGACGAG	Detection of insertion into *IS1* by Southern blot
JBN X28/X29	CACCCAAACCATCGTCCGC	CTGCGCAAGCCCTGCTTC	Check primers for transcription of *smrA*
JBN L39/L52	GAGGCGACGAGCAAGCTG	GTGCTCTCCAGGAGTCCG	Check primers for transcription of *hhtA* (*ANID_00733*)

Upper case letters indicate annealing nucleotides, lower case indicate tails for user cloning.

### RNA isolation and quantitative RT-PCR

RNA isolation from the *A. nidulans* strains and quantitative RT-PCR reactions were done as previously described in [13], except that disruption of biomass for RNA isolation was prepared with a Tissue-Lyser LT (Qiagen) by treating samples for 1 min at 45 mHz. The *A. nidulans* histone 3 encoding gene, *hhtA* (AN0733) was used as an internal standard for normalization of expression levels. All primers used for quantitative RT-PCR are shown in Table 3.

### Chemical characterization of mutant strains by UHPLC-DAD and LC-HRMS

All strains were grown as three point inoculations for 7 days at 37°C in the dark on solid glucose minimal, CYAs, RTO and YES media [35]. Extraction of metabolites was performed by the agar plug extraction method [38] using three 6 mm agar plugs/extract. Extracts were analyzed by UHPLC-DAD and LC-HRMS. UHPLC-DAD analysis was performed on a Dionex RSLC Ultimate 3000 (Dionex, Sunnyvale, CA) equipped with a diode-array detector. Separation was performed at 60°C on a 150 mm×2.1 mm ID, 2.6 µm Kinetex C_18_ column (Phenomenex, Torrence, CA) using a linear water/MeCN (both buffered with 50 ppm tri-fluoroacetic acid (TFA)) gradient starting from 15% MeCN to 100% over 7 min at a flow rate of 0.8 mL min^−1^. LC-HRMS analysis was performed on a MaXis 3G QTOF (Bruker Daltronics) coupled to a Dionex Ultimate 3000 UHPLC system equipped with a 100×2.0 mm, 2.6 µm, Kinetex C-18 column. The separation column was held at a temperature of 40°C and a gradient system composed of A: 20 mM formic acid in water, and B: 20 mM formic acid in acetonitrile was used. The flow was 0.4 ml/min, 85% A graduating to 100% B in 0–10 min, 100% B 10–13 min, 85% A 13.1–15 min. For calibration, a mass spectrum of sodium formate was recorded at the beginning of each chromatogram using a divert valve (0.3–0.4 min). Samples were analyzed both in positive and negative ionization mode. De-replication of induced compounds were performed by comparison of accurate mass to the metabolite database Antibase2009 [39], comparison of UV spectra to published data as well as authentic standards (JH-III, Sigma Aldrich).

### Chemical characterization of mutant strains by GC-MS

Volatile metabolites were collected during days 5–7 for the strains inoculated in CYAs. To collect the volatiles, a stainless steel Petri dish lid with a standard 1/4 Swagelock™ replaced the usual lid [40]. This lid possessed a standard 1/4 Swagelok fitting with PTFE insert in the centre that is used to hold a charcoal tube (SKC, 226-01). The collected volatiles were extracted from the charcoal tube with 0.3 mL of ether (Sigma Aldrich). The samples were concentrated to approximately 0.1 mL using a nitrogen flow in a GC vial and analysed using a Finnigan Focus GC coupled to a Finnigan Focus DSQ mass selective detector. The separation of the volatiles was done on a Supelco SLB™-5 MS capillary column, using He as carrier gas, at 1.2 mL/min. The injection and detection temperature was set to 220°C. One microlitre of each sample was injected into the GC–MS system. Chromatographic conditions were set to an initial temperature of 35°C for 1 min, raised at 6°C/min to 220°C and then 20°C/min to 260°C for 1 min. The separated compounds were characterized by their mass spectra generated by electron ionization (EI) at 70 eV at a scan range from *m*/*z* 35–300.

### Isolation of methyl (2E,6E)-10,11-dihydroxy-3,7,11-trimethyl-2,6-dodecadienoate (JH-diol)

NID477 was cultured on 100 CYAs plates for 7 days at 37°C in the dark. The plates were homogenized using a Stomacher homogenizer and 100 mL ethyl acetate (EtOAc) +1% formic acid (FA) pr. 10 plates. The extract was filtered after 1 hour and the remaining broth was extracted with EtOAc +1% FA for 24 hours. The extract was filtered and the two fractions pooled and dried down on a freeze drier. The crude extract was separated into three phases by dissolving it in 9∶1 MeOH:H_2_O – Milli-Q and extracted into a heptane phase followed by a dichloromethane (DCM) phase. The DCM phase was fractionated with a 10 g ISOL Diol column, using 13 steps of stepwise Hexane-dichloromethane-EtOAc-MeOH. JH-diol was present in the DCM fraction (9.5 mg) and was purified on a Waters HPLC W600/996PDA (Milford, MA, USA) using a RP column (Phenomenex Luna C18 (2), 250×10 mm, 5 µm, Torrance, CA, USA) using a gradient of 40% MeCN (H_2_O – Milli-Q (Millipore, MA, USA)) to 100% over 20 min with 50 ppm TFA and a flow of 4 mL/min. The fractions were concentrated on a rotarvap (Büchi V-855/R-215) and dried down under N_2_(g) to yield 2.0 mg of JH-diol. 1 and 2D NMR characterization (1H, DQF-COSY, H2BC, HMBC and HSQC) of the compound showed that the compound was a racemic mixture with a 2∶3 ratio of JH-diol a: JH-diol b. This is in agreement with an optical rotation of 0.0. The chemical shifts differed most in the reduced end of JH-diol, where the stereocenter is present, whereas the chemical shifts from C5 to C1 were overlaying. The difference of chemical shifts of the two methyl groups (H_12_/C_12_ and H_13_/C_13_) and the two CH_2_ groups next to the stereocenter are due to the presence of the chiral center. The two diastereomers present in the JH-diol solution must be due to the presence of JH-diol in both the *E*- and *Z*-conformation at the C_6_ and C_7_ double bond. The carbon shifts are in good agreement with published data [26].

### Isolation of methyl (2E,6E)-10-hydroxy-11-formyl-3,7,11-trimethyl-2,6-dodecadienoate (compound 2)

Compound 2 was present in the 60∶40 DCM:EtOAc fraction (13.1 mg) of the Diol fractionation as described above and was purified on a Waters HPLC W600/996PDA (Milford, MA, USA) using a RP column (Phenomenex Luna C18(2), 250×10 mm, 5 µm, Torrance, CA, USA) using a gradient of 40% MeCN (H_2_O – Milli-Q (Millipore, MA, USA)) to 100% over 20 min. with 50 ppm TFA and a flow of 4 mL/min. The collections were concentrated on a rotarvap (Büchi V-855/R-215) and dried under N_2_(g) to yield 2.6 mg of compound 2.

### Isolation of methyl (2E,6E)-10,11-epoxid-3,7,11-trimethyl-2,6-dodecadienoate (JH III)

JH-III was present in the 46∶60 DCM:EtOAc fraction (26.2 mg) of the Diol fractionation as described for JH-diol and was purified on a Waters HPLC W600/996PDA (Milford, MA, USA) using a RP column (Phenomenex Luna C18(2), 250×10 mm, 5 µm, Torrance, CA, USA) using a gradient of 55% MeCN (H_2_O – Milli-Q (Millipore, MA, USA)) to 65% over 20 min. with 50 ppm TFA and a flow of 4 mL/min. The fractions were concentrated on a rotarvap (Büchi V-855/R-215) and dried under N_2_(g) to yield 1.4 mg of JH-III. However, the purified JH-III degraded before NMR experiments could be conducted. Instead, JH-III was identified based on comparison of accurate mass and retention time with authentic standard. HRMS (*m/z*): [M+H]^+^ calcd. for C_16_H_27_O_3_, 267.1955; found, 267.1957.; [M+Na]^+^ calcd. For C_16_H_26_O_3_Na, 289.1780; found, 289.1774.

### NMR studies and structure elucidation

NMR spectra were acquired in DMSO-d_6_ on a Varian Unity Inova 500 MHz spectrometer for JH-diol and JH-III and on a Bruker Avance 800 MHz spectrometer at the Danish Instrument Center for NMR Spectroscopy of Biological Macromolecules for compound 2 using standard pulse sequences. The spectra were referenced to this solvent with resonances δ_H_ = 2.49 and δ_C_ = 39.5.

### Characterization data of methyl (2E,6E)-10,11-dihydroxy-3,7,11-trimethyl-2,6-dodecadienoate (JH-diol)

NMR data for JH-diol-a: ^1^H NMR (500 MHz, DMSO-*d_6_*): δ 5.65 (s, 1 H), 5.07 (m, 1 H), 4.25 (d, J = 5.6 Hz, 1 H), 4.01 (s, 1 H), 3.57 (s, 3 H), 3.02 (ddd, J = 10.0, 5.6, 2.5 Hz, 1 H), 2.15 (m, 2 H), 2.15-2.11 (m, 2 H), 2.09 (s, 3 H), 1.87 (m, 2 H), 1.60 (m, 1 H), 1.56 (s, 3 H), 1.15 (m, 1 H), 1.02 (s, 3 H), 0.97 (s, 3 H); ^13^C NMR (125 MHz): δ 166.1, 159.7, 135.9, 122.2, 114.7, 76.6, 71.5, 50.4, 39.7, 36.2, 29.4, 26.1, 25.1, 24.2, 18.2, 15.7.

NMR data for JH-diol-b: ^1^H NMR (500 MHz, DMSO-*d_6_*): δ 5.65 (s, 1 H), 5.08 (m, 1 H), 3.74 (dd, J = 10.0, 3.0 Hz, 1 H), 3.57 (s, 3 H), 2.15 (m, 2 H), 2.15-2.11 (m, 2 H), 2.10 (m, 1 H), 2.09 (s, 3 H), 1.96 (m, 1 H), 1.57 (s, 3 H), 1.48 (m, 1 H), 1.41 (m, 1 H), 1.21 (s, 3 H), 1.08 (s, 3 H); ^13^C NMR (125 MHz): 166.1, 159.7, 134.9, 123.0, 114.7, 82.6, 79.4, 50.4, 39.7, 35.7, 29.2, 27.7, 25.1, 22.8, 18.2, 15.6. HRMS (*m/z*): [M+H]^+^ calcd. For C_16_H_29_O_4_, 285.2060; found, 285.2025; [M+Na]^+^ calcd. For C_16_H_28_O_4_Na, 307.1885; found, 307.1887; [α]_D_ = 0.0 (MeOH).

### Characterization data of methyl (2E,6E)-10-hydroxy-11-formyl-3,7,11-trimethyl-2,6-dodecadienoate (compound 2)

^1^H NMR (800 MHz, DMSO-*d_6_*): δ 8.23 (s, 1 H), 5.65 (q, J = 1.0 Hz, 1H), 5.05 (t, J = 6.9, 1 H), 4.62 (s, 1 H), 4.52 (d, J = 10.2 Hz, 1 H), 3.57 (s, 3 H), 2.15 (m, 2 H), 2.11 (m, 2 H), 2.09 (d, J = 1 Hz, 3 H), 1.93 (m, 1 H), 1.84 (m, 1 H), 1.75 (m, 1 H), 1.55 (s, 3 H), 1.46 (m, 1 H), 1.04 (s, 3 H), 1.03 (s, 3 H); ^13^C NMR (200 MHz): δ 166.2, 162.3, 159.9, 134.6, 123.4, 114.7, 79.7, 70.2, 50.4, 39.6, 35.7, 26.9, 25.2, 25.1, 25.1, 18.3, 15.6; HRMS (*m/z*): [M+H]^+^ calcd. for C_17_H_29_O_5_, 313.2010; found, 313.2010. [M+Na]^+^ calcd. for C_17_H_28_O_5_Na, 335.1828; found, 335.1831.

### Characterization data of methyl (2, 6, 10) -3,7,11-trimethyl-2,6-dodecadienoate (MF)

HRMS (*m/z*): [M+H]^+^ calcd. for C_16_H_27_O_2_, 251.2006; found, 251.2007.

### Confrontation with *D. melanogaster* larvae

Fungal strains were point-inoculated (1000 conidia in 1 µl Ringer solution) on 3 ml standard *Drosophila* medium [30] filled in 3.5 cm diameter Petri dishes. Prior to the transfer of ten sterile *D. melanogaster* larvae per plate, colonies were pre-incubated for two days at 25°C and constant darkness. Colonies were exposed to insects for four days. Subsequently, insects were removed and the plates snap frozen for metabolite profile analysis. Quantitative metabolite profile analyses were performed on groups of five biological replicates. Statistical analysis was performed with pair-wise comparisons using the student's t-test procedure. Evaluation of insect fitness followed the procedure described in Trienens et al. [30]. We confronted the larval stage of the fruit fly *Drosophila melanogaster* (wild type Oregon R strain) with the reference NID545 or the JH-producer strain NID477. Sterile two-day first-instar larvae were exposed to *A. nidulans* colonies growing on autoclaved standard *Drosophila* culture medium in 2 ml micro-tubes. There were N = 20 experimental units per fungal treatment and N = 10 mold-free control units. Insect developmental success was monitored in terms of (1) larva-to-pupa survival and development time, (2) emergence of flies, and (3) fly dry weight. Short development time and high body mass are considered to be positively correlated with fitness in *Drosophila* [41]. Experimental tubes were checked for pupae and emerged flies at about 2 p.m. each day for a total of 14 days after larval transfer. Emerged flies were removed from the tubes and stored deep-frozen. Subsequently, flies were lyophilized for 24 hours and the dry weight of all flies within each experimental unit was determined as a single value using a micro-balance.
